# Supplementary material for: Effects of Sun Withering Degree on Black Tea Quality Revealed via Non-Targeted Metabolomics
Source: Foods. 2023 Jun 20;12(12):2430. doi: 10.3390/foods12122430 (PMC10297470; doi:10.3390/foods12122430)
Supplement: Supplementary file 1 [file foods-12-02430-s001.zip › foods-2445032-supplementary.pdf]

Supplemental information：

Table S1. Experimental reagent information.

Table S2. Results of main environmental factors of black tea with different degrees of sun withering.

Table S3. Sensory evaluation results of black tea with different degrees of sun withering.

Table S4. Information on non-volatile compounds of black tea with different degrees of sun withering.

Table S5. Relative content of non-volatile compounds of black tea with different degrees of sun withering (mg/g).

Table S6. Correlation analysis between non-volatile components and sensory evaluation of black tea with different degrees of sun withering.

Table S7. Analysis results of the main physicochemical components of black tea with different degrees of sun withering (%).

Table S8. Relative content of volatile compounds in black tea with different degrees of sun withering (ug/g).

Figures S1. Aromatic amino acid content of black tea with different degrees of sun withering.

Figure S2. Key volatiles content of black tea with different degrees of sun withering.

Table S1. Experimental reagent information.

Reagents	Manufacturers	Purity	Catalogue number	Suppliers
L-Glutamine	Shanghai Yuanye Bio-Technology Co., Ltd	≥98%	B21917-200mg	Yuanye
L-Lysine	Shanghai Yuanye Bio-Technology Co., Ltd	≥98%	B21922-200mg	Yuanye
L-Arginine	Shanghai Yuanye Bio-Technology Co., Ltd	≥98%	B21920-200mg	Yuanye
L-Isoleucine	Shanghai Yuanye Bio-Technology Co., Ltd	≥98%	B21937-200mg	Yuanye
L-Leucine	Shanghai Yuanye Bio-Technology Co., Ltd	≥98%	B21925-200mg	Yuanye
L-Phenylalanine	Shanghai Yuanye Bio-Technology Co., Ltd	99%	S20063-25g	Yuanye
L-Tryptophan	Shanghai Yuanye Bio-Technology Co., Ltd	≥98%	B21930-200mg	Yuanye
L-Aspartic acid	Shanghai Yuanye Bio-Technology Co., Ltd	≥99%	B21934-200mg	Yuanye
L-Asparagine	Shanghai Yuanye Bio-Technology Co., Ltd	≥98%	B21935-200mg	Yuanye
L-Glutamic acid	Shanghai Yuanye Bio-Technology Co., Ltd	≥99%	B21916-200mg	Yuanye
L-Threonine	Shanghai Yuanye Bio-Technology Co., Ltd	≥98%	B21933-200mg	Yuanye
L-Proline	Shanghai Yuanye Bio-Technology Co., Ltd	99%	S20347-25g	Yuanye
L-Valine	Shanghai Yuanye Bio-Technology Co., Ltd	⩾98%	B21936-200mg	Yuanye
L-Tyrosine	Shanghai Yuanye Bio-Technology Co., Ltd	95%	S72728-25g	Yuanye
L-Methionin	Shanghai Yuanye Bio-Technology Co., Ltd	≥98%	B21913-200mg	Yuanye
L-Theamine	Shanghai Yuanye Bio-Technology Co., Ltd	⩾98%	B21912-20mg	Yuanye
Theaflavin	Shanghai Yuanye Bio-Technology Co., Ltd	≥95%	B20140-20mg	Yuanye
Theaflavin-3-gallate	Shanghai Yuanye Bio-Technology Co., Ltd	⩾98%	B20143-20mg	Yuanye
Theaflavin-3'-gallate	Shanghai Yuanye Bio-Technology Co., Ltd	⩾98%	B20142-20mg	Yuanye
Theaflavine-3,3'-digallate	Shanghai Yuanye Bio-Technology Co., Ltd	⩾98%	B50785-10mg	Yuanye

(-)-Gallocatechin/GC	Shanghai Yuanye Bio-Technology Co., Ltd	⩾98%	B30661-5mg	Yuanye
(-)-Epigallocatechin/EGC	Shanghai Yuanye Bio-Technology Co., Ltd	⩾98%	B20105-20mg	Yuanye
(+)-Catechin/C	Shanghai Yuanye Bio-Technology Co., Ltd	⩾98%	A10025-20mg	Yuanye
Epicatechin /EC	Shanghai Yuanye Bio-Technology Co., Ltd	99.4%	A10044-20mg	Yuanye
Epigallocatechin gallate/EGCG	Shanghai Yuanye Bio-Technology Co., Ltd	⩾98%	B20106-20mg	Yuanye
(-)-Gallocatechin gallate/GCG	Shanghai Yuanye Bio-Technology Co., Ltd	⩾98%	B20850-20mg	Yuanye
(-)-Epicatechin gallate/ECG	Shanghai Yuanye Bio-Technology Co., Ltd	⩾98%	B20103-20mg	Yuanye
Catechin gallate/CG	Shanghai Yuanye Bio-Technology Co., Ltd	⩾98%	B20350-20mg	Yuanye
Theobromine	Shanghai Yuanye Bio-Technology Co., Ltd	⩾98%	B73891-2mg	Yuanye
Theophylline	Shanghai Yuanye Bio-Technology Co., Ltd	⩾98%	B20144-50mg	Yuanye
Caffeine	Shanghai Yuanye Bio-Technology Co., Ltd	⩾95%	B81798-10mg	Yuanye
(-)-Epiafzelechin	Shanghai Yuanye Bio-Technology Co., Ltd	⩾98%	B30490-5mg	Yuanye
Kaempferide	Shanghai Yuanye Bio-Technology Co., Ltd	⩾98%	B21132-20mg	Yuanye
Quercetin	Shanghai Yuanye Bio-Technology Co., Ltd	⩾98%	B20527-20mg	Yuanye
Myricetin	Shanghai Yuanye Bio-Technology Co., Ltd	⩾98%	B21458-20mg	Yuanye
Vitexin	Shanghai Yuanye Bio-Technology Co., Ltd	98.5%	A10063-20mg	Yuanye
Isovitexin	Shanghai Yuanye Bio-Technology Co., Ltd	⩾98%	B21544-20mg	Yuanye
Quercitrin	Shanghai Yuanye Bio-Technology Co., Ltd	⩾98%	B20526-20mg	Yuanye
Astragaline	Shanghai Yuanye Bio-Technology Co., Ltd	⩾98%	B21704-20mg	Yuanye
Quercetin-7- O-α-L-rhamnoside	Shanghai Yuanye Bio-Technology Co., Ltd	⩾98%	B20530-20mg	Yuanye
Quercetin-7-O-β-D-glucopyranoside	Shanghai Yuanye Bio-Technology Co., Ltd	⩾98%	B20531-10mg	Yuanye
Hyperoside	Shanghai Yuanye Bio-Technology Co., Ltd	⩾98%	B20631-20mg	Yuanye
Isovitexin 2''-O-arabinoside	Shanghai Yuanye Bio-Technology Co., Ltd	⩾98%	B28111-10mg	Yuanye
Glucosyl-vitexin	Shanghai Yuanye Bio-Technology Co., Ltd	⩾98%	B50220-20mg	Yuanye
Quercetin-3-o-rutinose	Shanghai Yuanye Bio-Technology Co., Ltd	⩾98%	B50405-5mg	Yuanye

Procyanidin B1	Shanghai Yuanye Bio-Technology Co., Ltd	⩾95%	B21616-20mg	Yuanye
Procyanidin B2	Shanghai Yuanye Bio-Technology Co., Ltd	⩾98%	B21617-20mg	Yuanye
Quercetin-3-O-D-glucosyl]-(1-2)-L-rhamnoside	Shanghai Yuanye Bio-Technology Co., Ltd	⩾98%	B24587-10mg	Yuanye
Kaempferol	Shanghai Yuanye Bio-Technology Co., Ltd	⩾98%	B21126-20mg	Yuanye
Myricetin 3-O-galactoside	Shanghai Yuanye Bio-Technology Co., Ltd	⩾98%	B30932-5mg	Yuanye
Fumaric acid	Shanghai Yuanye Bio-Technology Co., Ltd	98%	B72471-1mg	Yuanye
α-Ketoglutaric acid	Shanghai Yuanye Bio-Technology Co., Ltd	≥98%	B24374-20mg	Yuanye
Shikimic acid	Shanghai Yuanye Bio-Technology Co., Ltd	⩾98%	B20838-20mg	Yuanye
Caffeic acid	Shanghai Yuanye Bio-Technology Co., Ltd	⩾98%	B20660-20mg	Yuanye
Chlorogenic acid	Shanghai Yuanye Bio-Technology Co., Ltd	≥98%	B20782-20mg	Yuanye
Gallic acid	Shanghai Yuanye Bio-Technology Co., Ltd	≥98%	B20851-20mg	Yuanye
Etofylline	Shanghai Yuanye Bio-Technology Co., Ltd	99%	S71039-25g	Yuanye
Cyclohexanone	Shanghai Yuanye Bio-Technology Co., Ltd	≥95%	B72470-100mg	Yuanye

Table S2. Results of main environmental factors of black tea with different degrees of sun withering.

Withering degree	Withering method	Withering time (h)			Temperature (°C)		Humidity (%)		Light intensity (Lux)	
		Withering time	Withering duration	Total time	Range	Average	Range	Average	Range	Average
CK	\	\	\	10.7	\	\	\	\	\	\
	Indoor	11:00--21:40	10.7		24~29	26.5	40~64	52.5	34~68	51.2
S72	sun	11:00--12:00	1.0	4.1	30~32	31.2	20~22	21.5	23420~24420	23920.4
	Indoor	12:00--15:05	3.1		24~27	26.3	40~64	52.5	34~68	51.4
S69	sun	11:00--12:10	1.2	4.2	30~32	31.2	20~22	21.5	23420~24420	23920.2
	Indoor	12:10--14:55	3.1		24~27	26.3	40~64	52.5	34~68	51.3
S66	sun	11:00--12:40	1.6	2.9	30~32	31.2	20~22	21.5	23420~24420	23402.1
	Indoor	12:40--14:35	1.3		24~27	26.3	40~64	52.5	34~68	51.1
S63	sun	11:00-- 12:50	1.8	3.0	30~32	31.2	20~22	21.5	22320~23420	22870.3

	Indoor	12:50--13:20	1.2		24~27	26.3	40~64	52.5	34~68	51.4
S60	sun	11:00-13:20	2.4	2.4	30~32	31.2	20~22	21.5	22320~23420	22870.4
	\	\	\		\	\	\	\	\	\

Table S3. Sensory evaluation results of black tea with different degrees of sun withering.

Withering degree	Appearance	Brew color	Aroma	Taste	Infused leaf
CK	brownish auburn	orange red bright	grass odour	slightly grassy and astringent	red and clear
S72	brownish auburn	red bright	slightly grass odour	slightly astringent	red and bright
S69	brownish auburn	red bright	pure sweet	Sweet and mellow	red and bright
S66	brownish auburn	red bright	sweet	Sweet and mellow	red and bright
S63	brownish auburn	red bright	slightly high	Sweet and mellow	red and bright
S60	brownish auburn	red bright	pure and normal	mellow	red and bright

Table S4. Information on non-volatile compounds of black tea with different degrees of sun withering.

NO	Compounds	Formula	Theoretical mass(m/z)	MS/MS fragments
	**Amino acids**			
1	L-Glutamine	$C_5H_{10}N_2O_3$	147.0764	130,84,56
2	L-Lysine	$C_6H_{14}N_2O_2$	147.1128	102,84,56
3	L-Arginine	$C_6H_{14}N_4O_2$	175.1190	158,130,116,70,60
4	L-Isoleucine	$C_6H_{13}NO_2$	132.1019	86,69,57
5	L-Leucine	$C_6H_{13}NO_2$	132.1019	86,69,55
6	L-Phenylalanine	$C_9H_{11}NO_2$	166.0863	120,103,77,51
7	L-Tryptophan	$C_{11}H_{12}N_2O_2$	205.0972	188,170,146,118
8	L-Aspartic acid	$C_4H_7NO_4$	134.0448	88,74,61
9	L-Asparagine	$C_4H_8N_2O_3$	133.0608	87,74,70,60
10	L-Glutamic acid	$C_5H_9NO_4$	148.0604	130,102,84,56
11	L-Threonine	$C_4H_9NO_3$	120.0655	94,74,56
12	L-Proline	$C_5H_9NO_2$	116.0706	70
13	L-Valine	$C_5H_{11}NO_2$	118.0863	72,55
14	L-Tyrosine	$C_9H_{11}NO_3$	182.0812	136,123,119,95,91,27
15	L-Methionin	$C_5H_{11}NO_2S$	150.0583	56, 61, 74, 87, 104, 115, 133
16	L-Theamine	$C_7H_{14}N_2O_3$	175.1077	158,129,84,56

	**Theaflavins**			
17	Theaflavin	$C_{29}H_{24}O_{12}$	565.1341	427,277,139
18	Theaflavin-3-gallate	$C_{36}H_{28}O_{16}$	717.145	579,379,277,139
19	Theaflavin-3'-gallate	$C_{36}H_{28}O_{16}$	717.145	591,579,333,277,139
20	Theaflavine-3,3'-digallate	$C_{43}H_{32}O_{20}$	869.156	743,699,529,333,277,000
	**Catechins**			
21	(-)-gallocatechin/GC	$C_{15}H_{14}O_7$	307.0812	223,195,163,139,95
22	(-)-Epigallocatechin/EGC	$C_{15}H_{14}O_7$	307.0812	223,195,163,139,95
23	(+)-Catechin/C	$C_{15}H_{14}O_6$	291.0863	207,139,123,95,55
24	Epigallocatechin gallate/EGCG	$C_{22}H_{18}O_{11}$	459.0922	289,205,181,153,139,123,65
25	Epicatechin /EC	$C_{15}H_{14}O_6$	291.0863	207,139,123,95,55
26	(-)-Gallocatechin gallate/GCG	$C_{22}H_{18}O_{11}$	459.0922	289,205,181,153,139,123,65
27	(-)-Epicatechin gallate/ECG	$C_{22}H_{18}O_{10}$	443.0973	291,273,207,153,139,77
28	Catechin gallate/CG	$C_{22}H_{18}O_{10}$	443.0973	291,273,207,153,139,77
	**Alkaloids**			
29	Theobromine	$C_7H_8N_4O_2$	181.072	163,138,122,110,83,69,67,56
30	Theophylline	$C_7H_8N_4O_2$	181.072	124,96,69
31	Caffeine	$C_8H_{10}N_4O_2$	195.0877	138,123,110,83,69,56
	**Flavonoid glycosides**			
32	(-)-Epiafzelechin	$C_{15}H_{14}O_5$	275.0914	257,233,191,163,149,139,121,107,95,55
33	Kaempferol	$C_{15}H_{10}O_6$	287.0550	153,69
34	Kaempferide	$C_{16}H_{12}O_6$	301.0707	286,258,230,153,69
35	Quercetin	$C_{15}H_{10}O_7$	303.0499	229,153,69
36	Myricetin	$C_{15}H_{10}O_8$	319.0448	273,245,217,165,153,137,111,69
37	Vitexin	$C_{21}H_{20}O_{10}$	433.1129	415,397,367,313,283
38	Isovitexin	$C_{21}H_{20}O_{10}$	433.1129	416,380,350,338,314,296,284,272,244.165,121
39	Quercitrin	$C_{21}H_{20}O_{11}$	449.1078	303,129,85,71,57
40	Astragaline	$C_{21}H_{20}O_{11}$	449.1074	288,259,214,153,127,85
41	Quercetin-7- O-α-L-rhamnoside	$C_{21}H_{20}O_{11}$	449.1078	303,229,85
42	Quercetin-7-O-β-D-glucopyranoside	$C_{21}H_{20}O_{12}$	465.1028	303
43	Hyperoside	$C_{21}H_{20}O_{12}$	465.1025	404,365,335,298,266,232,203,153,113,81
44	Myricetin 3-O-galactoside	$C_{21}H_{20}O_{13}$	481.0977	319,127,85,61
45	Isovitexin 2''-O-arabinoside	$C_{26}H_{28}O_{14}$	565.1552	435,369,314,115,73
46	Procyanidin B1	$C_{30}H_{26}O_{12}$	579.1497	513,451,411,374,330,289,260,164,127,90
47	Procyanidin B2	$C_{30}H_{26}O_{12}$	579.1497	537,411,287,247,205

48	Glucosyl-vitexin	$C_{27}H_{30}O_{16}$	611.1607	449,329,287,85
49	Quercetin-3-o-rutinose	$C_{27}H_{30}O_{16}$	611.1607	449,329,287,85
50	Quercetin-3-O-D-glucosyl-(1-2)-L-rhamnoside	$C_{27}H_{30}O_{16}$	611.1607	449,431,413,369,345,303,129,85
	**Organic acids**			
51	Fumaric acid	$C_4H_4O_4$	117.0182	99,75,71,59,58,53
52	α-Ketoglutaric acid	$C_5H_6O_5$	147.0288	84
53	Shikimic acid	$C_7H_{10}O_5$	175.0601	158,84,56
54	Caffeic acid	$C_9H_8O_4$	181.0495	163,145,135,117,107,89,79
55	Chlorogenic acid	$C_{16}H_{18}O_9$	355.1042	163,145,135,117,89
56	Gallic acid	$C_7H_6O_5$	171.0288	153,135,127,125,109,107,81,53
	**GVBs**			
57	(Z)-3-hexenyl β-D-glucoside	$C_{12}H_{22}O_6$	285.1309	268,235,211,179,153,129,105,92
58	Benzyl β-D-glucoside	$C_{13}H_{18}O_6$	293.0996	265,234,205,184,169,143,129,117
59	2-Phenylethyl β-D-glucoside	$C_{14}H_{20}O_6$	307.1152	292,266,232,215,201,185,165,144,126,102,85
60	Geranyl β-D-glucoside	$C_{16}H_{28}O_6$	339.1778	301,262,236,203,179,164,117
61	Nerol β-D-glucoside	$C_{16}H_{28}O_6$	339.1778	292,267,237,220,203,168,143,121,96,81
62	Benzyl β-D-primeveroside	$C_{18}H_{26}O_{10}$	425.1418	404,330,293,231,200,150,119,95
63	2-Phenylethyl β-D-primeveroside	$C_{19}H_{28}O_{10}$	439.1575	401,307,275,218,170,123
64	Geranyl β-D-primeveroside	$C_{21}H_{36}O_{10}$	471.2201	335,275,245,203,169,123
65	Nerol β-D-primeveroside	$C_{21}H_{36}O_{10}$	471.2201	426,381,335,275,203,173,141,105,77

Table S5. Relative content of non-volatile compounds of black tea with different degrees of sun withering (mg/g).

NO	Compounds	CK	S72	S69	S66	S63	S60
	**Amino acids**						
1	L-Glutamine	0.3212±0.012b	0.3232±0.009b	0.3305±0.004b	0.3325±0.001b	0.3223±0.003b	0.3562±0.012a
2	L-Lysine	0.0711±0.003b	0.0627±0.001c	0.066±0.001c	0.0716±0.001b	0.0637±0.001c	0.0804±0.003a
3	L-Arginine	1.2863±0.092d	1.6349±0.13c	2.6615±0.211a	2.3806±0.122ab	2.2082±0.27b	2.3066±0.027b
4	L-Isoleucine	0.3766±0.021a	0.2245±0.015c	0.2005±0.004d	0.2607±0.016b	0.2333±0.01c	0.2712±0.007b
5	L-Leucine	2.1676±0.029a	1.5694±0.036e	1.5902±0.022de	1.7061±0.025c	1.6321±0.005d	2.0802±0.016b
6	L-Phenylalanine	3.2465±0.12a	0.8879±0.011b	0.8962±0.011b	0.7097±0.014c	0.5037±0.006d	0.4927±0.007d
7	L-Tryptophan	0.5638±0.026a	0.3845±0.012c	0.3827±0c	0.3851±0.004c	0.3656±0.004c	0.4244±0.017b
8	L-Aspartic acid	0.008±0b	0.0047±0e	0.0053±0d	0.0061±0c	0.0058±0c	0.0096±0a
9	L-Asparagine	0.1471±0.008a	0.1062±0.003cd	0.0991±0.004d	0.1099±0.002c	0.1128±0c	0.1364±0.003b

10	L-Glutamic acid	0.0335±0.001d	0.035±0.001c	0.0363±0b	0.0364±0.001b	0.0333±0d	0.0395±0a
11	L-Threonine	0.3214±0.027a	0.0943±0.007b	0.0903±0.002bc	0.0736±0.003cd	0.0576±0.002d	0.0536±0.004d
12	L-Proline	0.023±0.001d	0.0265±0b	0.0267±0.001b	0.028±0a	0.0253±0.001c	0.0276±0.001ab
13	L-Valine	0.2022±0.002d	0.2192±0.008bc	0.2134±0.01c	0.2315±0.002a	0.2289±0.003ab	0.1495±0.006e
14	L-Tyrosine	0.332±0.011a	0.3022±0.006d	0.3064±0.008cd	0.3158±0.007bc	0.2987±0.005d	0.3287±0.007ab
15	L-Methionin	0.0004±0d	0.0006±0b	0.0006±0b	0.0009±0a	0.0004±0d	0.0005±0c
16	L-Theanine	8.7002±0.276c	8.7094±0.066c	9.0963±0.128b	9.0531±0.144b	8.4244±0.08d	9.5482±0.077a
	**Total**	17.8009±0.437a	14.5849±0.137d	16.002±0.356bc	15.7018±0.305c	14.5161±0.344d	16.3053±0.079b
	**Theaflavins**						
17	Theaflavin	0.1734±0.003f	0.253±0.003b	0.2763±0.003a	0.2439±0.003c	0.2185±0.001d	0.2112±0.003e
18	Theaflavin-3-gallate	0.1537±0.004d	0.1933±0.001b	0.2114±0.002a	0.1955±0.003b	0.1743±0.001c	0.1769±0.003c
19	Theaflavin-3'-gallate	0.1218±0.004d	0.1679±0.004b	0.1905±0.002a	0.1729±0.005b	0.149±0.001c	0.1559±0.006c
20	Theaflavine-3,3'-digallate	0.1539±0.005e	0.1791±0.001c	0.1916±0.002a	0.1898±0.002ab	0.1727±0.001d	0.1867±0.003b
	**Total**	0.6028±0.013d	0.7933±0.002b	0.8698±0.007a	0.802±0.013b	0.7146±0.003c	0.7307±0.014c
	**Catechins**						
21	(-)-gallocatechin/GC	0.0321±0.001e	0.074±0.004b	0.0989±0.001a	0.0674±0.002c	0.0471±0.001d	0.0328±0.001e
22	(-)-Epigallocatechin/EGC	0.0538±0.003f	0.1166±0.003b	0.1552±0.003a	0.1089±0.003c	0.0793±0.002d	0.0606±0.001e
23	(+)-Catechin/C	0.0934±0.004e	0.1873±0.003b	0.2237±0.004a	0.1498±0.007c	0.1286±0.001d	0.0956±0.004e
24	Epigallocatechin gallate/EGCG	0.0771±0.004e	0.1702±0.008b	0.2126±0.009a	0.1373±0.006c	0.115±0.003d	0.0598±0.002f
25	Epicatechin /EC	0.1502±0.011e	0.2993±0.013b	0.3588±0.001a	0.2456±0.006c	0.2049±0.001d	0.1633±0.003e
26	(-)-Gallocatechin gallate/GCG	0.1307±0.001e	0.2836±0.01b	0.3536±0.012a	0.2244±0.01c	0.1879±0.001d	0.111±0.005f
27	(-)-Epicatechin gallate/ECG	0.1561±0.003e	0.279±0.006b	0.3238±0.003a	0.2198±0.007c	0.1897±0.001d	0.1191±0.005f
28	Catechin gallate/CG	0.1561±0.003e	0.279±0.006b	0.3238±0.003a	0.2198±0.007c	0.1897±0.001d	0.1191±0.005f
	**Total**	0.8496±0.022e	1.6889±0.039b	2.0504±0.023a	1.3729±0.041c	1.1421±0.004d	0.7612±0.014f
	**Alkaloids**						
29	Theobromine	0.2058±0.003d	0.3129±0.001c	0.3196±0.005bc	0.3343±0.005ab	0.3253±0.011c	0.3536±0.025a
30	Theophylline	0.3202±0.009e	0.4817±0.011d	0.5122±0.012c	0.5592±0.011b	0.5471±0.009b	0.6497±0.015a
31	Caffeine	22.9411±0.302b	22.5237±0.119b	22.7139±0.164b	22.8528±0.073b	21.9175±0.087c	23.4493±0.459a
	**Total**	23.4671±0.296b	23.3184±0.131b	23.5458±0.171b	23.7463±0.062b	22.7898±0.083c	24.4526±0.494a
	**Flavonoid glycosides**						
32	(-)-Epiafzelechin	1.1904±0.027e	1.4305±0.01c	1.4556±0.005c	1.5132±0.015b	1.3198±0.009d	1.6296±0.02a
33	Kaempferol	0.0415±0.001a	0.0378±0.001c	0.0388±0b	0.0392±0b	0.0394±0b	0.0386±0.001bc
34	Kaempferide	0.6603±0.03d	0.8655±0.027c	1.0831±0.1b	1.2026±0.02a	0.536±0.028e	0.6256±0.022d
35	Quercetin	0.2246±0.006bc	0.2404±0a	0.2247±0.001bc	0.2303±0.005b	0.2204±0.003c	0.2269±0.003b
36	Myricetin	0.2104±0.006bc	0.2268±0.012a	0.2173±0.005abc	0.2178±0.007abc	0.2101±0.005c	0.2224±0.004ab

37	Vitexin	0.0083±0d	0.0096±0b	0.0094±0b	0.0096±0b	0.0101±0a	0.0088±0c
38	Isovitexin	0.0076±0b	0.0089±0a	0.0088±0a	0.0091±0a	0.0092±0a	0.0075±0b
39	Quercitrin	0.0617±0.002bc	0.0617±0.002bc	0.0616±0.001bc	0.0631±0.001b	0.0595±0.001c	0.0667±0.001a
40	Astragaline	0.4485±0.004c	0.4515±0.007c	0.4552±0.006bc	0.4636±0.001b	0.4355±0.008d	0.4858±0.004a
41	Quercetin-7- O-α-L-rhamnoside	0.3282±0.002cd	0.335±0.005bc	0.3372±0.004b	0.3407±0.002b	0.3249±0.005d	0.3564±0.003a
42	Quercetin-7-O-β-D-glucopyranoside	0.0897±0.001e	0.1017±0.002b	0.106±0.001a	0.101±0.001bc	0.0973±0d	0.0992±0.001cd
43	Hyperoside	0.0862±0.001e	0.1034±0.001b	0.1058±0.002a	0.0965±0.001c	0.0977±0.002c	0.0935±0d
44	Myricetin 3-O-galactoside	0.0092±0.001b	0.008±0c	0.0089±0b	0.0079±0.001c	0.0077±0c	0.0103±0a
45	Isovitexin 2''-O-arabinoside	0.1734±0.003f	0.253±0.003b	0.2768±0.003a	0.2439±0.003c	0.2185±0.001d	0.2112±0.003e
46	Procyanidin B1	0.0831±0.003c	0.1019±0.005b	0.1161±0.001a	0.1053±0.002b	0.0414±0.002d	0.0306±0.001e
47	Procyanidin B2	0.4193±0.024d	0.5335±0.021c	0.8447±0.053b	0.8978±0.023a	0.3524±0e	0.3303±0.021e
48	Glucosyl-vitexin	0.0377±0e	0.0476±0.001b	0.0494±0a	0.0432±0.001c	0.0442±0.001c	0.0407±0d
49	Quercetin-3-o-rutinose	0.0421±0.001e	0.0525±0.001b	0.0542±0a	0.048±0.001c	0.0483±0c	0.0455±0d
50	Quercetin-3-O-D-glucosyl-(1-2)-L-rhamnoside	0.002±0c	0.0021±0bc	0.002±0bc	0.0022±0ab	0.0022±0a	0.0021±0abc
	**Total**	4.1242±0.066e	4.8716±0.029c	5.4555±0.113b	5.635±0.028a	4.0747±0.036e	4.5318±0.05d
	**Organic acids**						
51	Fumaric acid	0.0199±0.001d	0.0216±0bc	0.0226±0.001ab	0.0235±0.001a	0.0209±0.001cd	0.0237±0.001a
52	α-Ketoglutaric acid	0.3302±0.005b	0.3238±0.009b	0.3274±0.009b	0.3308±0.004b	0.3284±0.005b	0.3648±0.004a
53	Shikimic acid	8.7002±0.276c	8.7094±0.066c	9.1002±0.131b	9.103±0.078b	8.4287±0.081d	9.5482±0.077a
54	Caffeic acid	0.3188±0.01e	0.4817±0.011d	0.5122±0.012c	0.5591±0.011b	0.5471±0.009b	0.9968±0.02a
55	Chlorogenic acid	0.1566±0.001a	0.1557±0.001a	0.1556±0.001a	0.1475±0.001b	0.1426±0c	0.1356±0.002d
56	Gallic acid	0.0392±0e	0.062±0.001b	0.0692±0.001a	0.0626±0.002b	0.0586±0c	0.0564±0d
	**Total**	9.5649±0.26c	9.7542±0.081c	10.1871±0.123b	10.2264±0.072b	9.5261±0.078c	11.1256±0.103a
	**GVBs**						
57	(Z)-3-hexenyl β-D-glucoside	0.0638±0.003d	0.077±0.005c	0.0688±0.003d	0.1054±0.002b	0.1171±0.005a	0.1171±0.005a
58	Benzyl β-D-glucoside	0.0376±0.001d	0.0549±0.001b	0.0581±0.001a	0.0571±0a	0.0511±0.001c	0.0526±0.001c
59	2-Phenylethyl β-D-glucoside	0.0584±0.004d	0.1166±0.003b	0.1586±0.005a	0.1079±0.004c	0.0601±0.001d	0.0601±0.001d
60	Geranyl β-D-glucoside	0.297±0.005b	0.2933±0.006b	0.2991±0.003b	0.2968±0.001b	0.3277±0.004a	0.3277±0.004a
61	Nerol β-D-glucoside	0.1491±0.013d	0.2±0.001c	0.2085±0.012c	0.2447±0.01b	0.2827±0.005a	0.2827±0.005a
62	Benzyl β-D-primeveroside	0.0023±0c	0.0033±0a	0.0019±0d	0.003±0b	0.0015±0e	0.0015±0e
63	2-Phenylethyl β-D-primeveroside	0.0601±0.001d	0.1016±0.002b	0.109±0.002a	0.0932±0.002c	0.0568±0.001e	0.0568±0.001e
64	Geranyl β-D-primeveroside	0.2473±0.003b	0.2547±0.003a	0.2554±0.006a	0.2537±0.001a	0.2526±0.002ab	0.2506±0.002ab
65	Nerol β-D-primeveroside	0.1617±0.001b	0.1659±0.003a	0.1655±0.003a	0.1636±0.001ab	0.1645±0.001ab	0.1552±0.002c
	**Total**	1.0773±0.014c	1.2675±0.006b	1.3249±0.024a	1.3254±0.004a	1.3141±0.007a	1.3042±0.009a
	**All total**	57.198±0.963b	56.2788±0.308b	59.4356±0.221a	58.8098±0.371a	54.0578±0.408c	59.2113±0.739a

Table S6. Correlation analysis between non-volatile components and sensory evaluation of black tea with different degrees of sun withering.

NO	Compounds	Taste score	Total score
	**Amino acids**		
1	L-Glutamine	0.058	0.030
2	L-Lysine	-0.176	-0.225
3	L-Arginine	0.863**	0.843**
4	L-Isoleucine	-0.692**	-0.694**
5	L-Leucine	-0.645**	-0.672**
6	L-Phenylalanine	-0.640**	-0.611**
7	L-Tryptophan	-0.713**	-0.705**
8	L-Aspartic acid	-0.428	-0.477*
9	L-Asparagine	-0.723**	-0.752**
10	L-Glutamic acid	0.188	0.158
11	L-Threonine	-0.644**	-0.617**
12	L-Proline	0.563*	0.549*
13	L-Valine	0.389	0.437
14	L-Tyrosine	0.650**	0.632**
15	L-Methionine	0.484*	0.523*
16	L-Theanine	0.141	0.120
	**Theaflavins**		
17	Theaflavin	0.707**	0.737**
18	Theaflavin-3-gallate	0.739**	0.766**
19	Theaflavin-3'-gallate	0.762**	0.779**
20	Theaflavine-3,3'-digallate	0.615**	0.609**
	**Catechins**		
21	(-)-gallocatechin/GC	0.644**	0.691**
22	(-)-Epigallocatechin/EGC	0.665**	0.711**
23	(+)-Catechin/C	0.564*	0.611**
24	Epigallocatechin gallate/EGCG	0.578*	0.628**
25	Epicatechin /EC	0.579*	0.626**
26	(-)-Gallocatechin gallate/GCG	0.570*	0.617**
27	(-)-Epicatechin gallate/ECG	0.498*	0.551*
28	Catechin gallate/CG	0.498*	0.551*
	**Alkaloids**		

29	Theobromine	0.621**	0.586*
30	Theophylline	0.523*	0.476*
31	Caffeine	-0.248	-0.259
	**Flavonoid glycosides**		
32	(-)-Epiafzelechin	0.391	0.371
33	Kaempferol	-0.305	-0.314
34	Kaempferide	0.566*	0.624**
35	Quercetin	-0.258	-0.207
36	Myricetin	-0.078	-0.051
37	Vitexin	0.622**	0.625**
38	Isovitexin	0.675**	0.694**
39	Quercitrin	-0.106	-0.139
40	Astragaline	-0.012	-0.045
41	Quercetin-7- O-α-L-rhamnoside	0.075	0.040
42	Quercetin-7-O-β-D-glucopyranoside	0.713**	0.722**
43	Hyperoside	0.606**	0.624**
44	Myricetin 3-O-galactoside	-0.302	-0.342
45	Isovitexin 2"-O-arabinoside	0.707**	0.737**
46	Procyanidin B1	0.252	0.328
47	Procyanidin B2	0.664**	0.718**
48	Glucosyl-vitexin	0.601**	0.625**
49	Quercetin-3-o-rutinose	0.583*	0.608**
50	Quercetin-3-O-D-glucosyl-(1-2)-L-rhamnoside	0.374	0.358
	**Organic acids**		
51	Fumaric acid	0.468*	0.445
52	α-Ketoglutaric acid	-0.154	-0.208
53	Shikimic acid	0.162	0.143
54	Caffeic acid	0.131	0.073
55	Chlorogenic acid	-0.152	-0.082
56	Gallic acid	0.795**	0.804**
	**GVBs**		
57	(Z)-3-hexenyl β-D-glucoside	0.291	0.234
58	Benzyl β-D-glucoside	0.760**	0.765**
59	2-Phenylethyl β-D-glucoside	0.564*	0.615**

60	Geranyl β-D-glucoside	0.095	0.013
61	Nerol β-D-glucoside	0.463	0.405
62	Benzyl β-D-primeveroside	-0.173	-0.102
63	2-Phenylethyl β-D-primeveroside	0.449	0.511*
64	Geranyl β-D-primeveroside	0.562*	0.571*
65	Nerol β-D-primeveroside	0.327	0.374

Table S7. Analysis results of the main physicochemical components of black tea with different degrees of sun withering (%).

Withering degree	Tea Polyphenols	Free amino acids	Soluble sugars	Theaflavins	Thearubins	Theabrownins
CK	10.14±0.09c	1.76±0.03b	3.00±0.03b	0.34±0.04c	5.29±0.03c	7.96±0.03a
S72	11.34±0.09a	1.83±0.02a	2.97±0.02b	0.44±0.01b	5.86±0.06ab	7.39±0.02d
S69	11.34±0.09a	1.83±0.03a	2.99±0.05b	0.52±0.03a	6.01±0.13a	7.19±0.02e
S66	11.33±0.15a	1.84±0.03a	2.99±0.01b	0.50±0.02a	5.94±0.07a	7.69±0.04c
S63	11.07±0.05b	1.83±0.04a	2.98±0.04b	0.45±0.01b	5.91±0.07a	7.64±0.09c
S60	11.03±0.07b	1.85±0.05a	3.07±0.01a	0.35±0.03c	5.72±0.15b	7.82±0.07b

Table S8. Relative content of volatile compounds in black tea with different degrees of sun withering (ug/g).

NO.	Compound name	CAS	Odor	CK	S72	S69	S66	S63	S60
				**Alcohols**					
1	Benzyl alcohol	100-51-6	boiled, cherries	23.316±2.345a	21.743±0.307a	23.697±1.032a	23.861±0.603a	22.806±1.194a	22.492±1.86a
2	(Z)-linalool oxide (furanoid)	5989-33-3	floral	23.687±1.44d	24.428±1.385d	30.463±0.04a	27.485±0.882b	26.414±0.738cb	25.138±0.582cd
3	(E)-linalool oxide (furanoid)	34995-77-2	floral	33.578±2.446bc	30.638±2.808c	36.97±2.336a	35.676±0.399ab	33.609±0.074bc	32.142±0.797c
4	Linalool	78-70-6	coriander, floral	35.702±1.901bc	35.925±1.524bc	42.147±3.671a	39.732±1.186ab	42.051±4.301a	33.811±3.014c
5	Dehydrolinalool	29957-43-5	mouldy	26.698±1.6c	30.677±2.458b	35.269±1.952a	33.216±0.329ab	32.468±1.519ab	25.806±0.544c

6	Benzeneethanol	60-12-8	fruit, honey	22.586±1.027e	31.886±0.295b	34.212±0.312a	32.367±0.259b	30.219±1.587c	26.794±0.27d
7	(E)-linalool oxide (pyranoid)	39028-58-5	woody, fresh	24.96±1.52a	18.695±1.675	20.368±3.251	20.24±2.116	19.679±2.599	19.478±0.152
8	(Z)-linalool oxide (pyranoid)	14009-71-3	citrus, green	38.089±1.863a	32.945±3.518b	36.735±4.465ab	39.975±1.052a	35.517±4.771ab	33.879±0.632b
9	α-Terpineol	10482-56-1	lilac, floral	0.824±0.045a	0.623±0.012c	0.626±0.009c	0.726±0.063b	0.692±0.004b	0.572±0.019c
10	Nopol	128-50-7	sweet, balsamic	2.276±0.049c	2.182±0.146c	2.502±0.118b	2.233±0.136c	2.812±0.081a	2.279±0.115c
11	Nerol	106-25-2	floral, fruit	7.709±0.585a	6.609±0.493b	6.387±0.172b	6.405±0.279b	6.313±0.238b	5.043±0.504c
12	Geraniol	106-24-1	geranium, lemon, peel	72.019±1.221ab	73.085±2.101ab	76.194±4.477a	71.301±1.854bc	70.24±0.667bc	66.547±3.707c
13	Cuminol	536-60-7	herb, wood	2.668±0.181a	1.738±0.275b	1.667±0.043b	1.237±0.059c	1.368±0.139bc	1.142±0.125c
14	2-Hexyl-1-decanol	2425-77-6	null	0±0d	0±0d	0±0d	0.102±0.024c	0.191±0.014b	0.227±0.028a
15	3,7,11-trimethyldodecan-1-ol	6750-34-1	null	0.429±0.019a	0.301±0.027c	0.456±0.018a	0.448±0.014a	0.371±0.031b	0.38±0.008b
16	Nerolidol	40716-66-3	floral, green, citrus	9.796±0.195a	5.235±0.176c	7.466±0.233b	4.003±0.243d	4.016±0.132d	3.561±0.073e
17	2,6-dimethyl-3,7-octadien-2,6-diol	51276-34-7	null	0±0b	0±0b	0±0b	0±0b	0±0b	1.145±0.027a
18	2,6-dimethylocta-1,7-diene-3,6-diol	51276-33-6	null	2.925±0.321a	2.252±0.235bc	2.485±0.363abc	2.688±0.335ab	2.095±0.135c	0±0d
19	(E)-para-2,8-Menthadien-1-ol	7212-40-0	fresh, minty	0.56±0.013a	0.421±0.016c	0.486±0.013b	0.406±0.003c	0.335±0.01d	0.266±0.003e
20	(E)-para-2-menthen-1-ol	29803-81-4	null	0±0e	0.818±0.029a	0.941±0.11a	0.76±0.07b	0.667±0.014c	0.619±0.021d
21	Laevo-pinocarveol	547-61-5	woody, balsamic, fennel	0±0c	0.227±0.032b	0.371±0.046a	0±0c	0±0c	0±0c
22	(Z)-verbenol	1845-30-3	null	0±0c	1.188±0.082a	1.018±0.067b	0±0c	0±0c	0±0c
23	(E,Z)-3,6-Nonadien-1-ol	56805-23-3	green, cucumber	0.541±0.034a	0.47±0.021bc	0.466±0.009bc	0.507±0.034ab	0.444±0.028c	0.424±0.038c
24	Dihydrocarveol	38049-26-2	null	0.985±0.051b	1.276±0.009a	0.775±0.011c	0.683±0.017d	0.663±0.04d	0.395±0.003e
25	α-cadinol	481-34-5	herb, wood	0.359±0.017a	0.224±0.007d	0.274±0.003b	0.256±0.011c	0.165±0.009e	0.179±0.016e

26	Myrtenol	19894-97-4	woody, pine, balsam	0±0b	0±0b	0±0b	0±0b	0±0b	0.073±0.002a
27	3-methyl-3-buten-2-ol	10473-14-0	null	0.136±0.015a	0.135±0.003a	0.136±0.01a	0.088±0.008b	0.073±0.002c	0.059±0.006d
28	Cis-2-penten-1-ol	1576-95-0	green, ethereal, medicinal	0.405±0.033b	0.369±0.01c	0.427±0.03ab	0.515±0.071a	0.406±0.019b	0.252±0.039d
29	2-Hexanol	626-93-7	winey, fruity, fatty	0.029±0c	0.129±0.011a	0.112±0.008a	0.115±0.017a	0.034±0.005c	0.052±0b
30	2,3-dimethylpentan-1-ol	10143-23-4	null	0.581±0.015bc	0.637±0.001ab	0.679±0.038a	0.632±0.075ab	0.531±0.056c	0.218±0.003d
31	2,3-dimethylbutan-1-ol	19550-30-2	null	1.482±0.097c	2.121±0.262a	1.683±0.032b	1.743±0.081b	0.192±0.016a	0.166±0.009b
32	2,5-dimethyl-2-hexanol	3730-60-7	null	1.461±0.141b	0.501±0.017c	2.294±1.698a	0.163±0.008e	0.295±0.005d	0.174±0.016e
33	(Z)-hex-3-en-1-ol	928-96-1	grass, green, fruit	3.934±0.314b	4.848±0.186a	3.268±0.529b	2.652±0.072c	2.575±0.143c	2.152±0.179d
34	Trans-2-Hexen-1-ol	928-95-0	fresh, green, fruity	0.233±0.003b	0.169±0.008c	0.223±0.008b	0.287±0.036a	0.226±0.017b	0.166±0.012c
35	Hexanol	111-27-3	banana, flower, grass	0.182±0.023cd	0.166±0.001d	0.233±0.018b	0.279±0.006a	0.288±0.015a	0.199±0.026bc
36	2-butoxyethanol	111-76-2	null	0.131±0.011a	0.15±0.013a	0.085±0.006b	0.061±0.004c	0.047±0.004d	0.04±0.007d
37	Trans-2,4-hexadien-1-ol	111-28-4	fruit	0.028±0.002c	0.08±0.004a	0.046±0.002b	0.041±0.001b	0.049±0.004b	0.047±0.007b
38	1-Octene-3-ol	3391-86-4	cucumber, earth, fat	0.062±0.001c	0.045±0.002d	0.049±0.004d	0.067±0.003b	0.072±0.002b	0.091±0.011a
39	(Z)-para-2,8-menthadien-1-ol	3886-78-0	null	0.101±0.001b	0.04±0e	0.08±0.001d	0.118±0.017a	0.123±0.003a	0.09±0.002c
40	Butyldiglycol	112-34-5	null	0.215±0.005c	0.217±0.012c	0.216±0.009c	0.296±0.032b	0.328±0.018b	0.426±0.03a
41	(Z)-2-(3,3-Dimethylcyclohexylidene)ethanol	26532-23-0	null	0.073±0a	0.041±0c	0.042±0.001c	0.073±0.001a	0.064±0.005b	0.054±0.006b
42	4,6,6-trimethylbicyclo[3.1.1]heptane-3,4-diol	53404-49-2	null	0.065±0.003c	0.037±0.002d	0.071±0.002b	0.084±0.005a	0.08±0.002a	0.085±0.011a
43	(E)-2-decen-1-ol	18409-18-2	fruit	0.004±0d	0.001±0e	0.075±0.001a	0.073±0.008a	0.051±0.001b	0.034±0.004c
44	2-butyl-1-octanol	3913-02-8	null	0.037±0.002d	0.15±0.017b	0.075±0.007c	0.125±0.016b	0.126±0.012b	0.333±0.024a
45	2-methyl undecanol	10522-26-6	balsam	0.029±0.002c	0.021±0.001d	0.035±0.005c	0.064±0.003b	0.068±0.001a	0.06±0.004b

46	(+)-Cedrol	77-53-2	cedarwood, woody, sweet	0.046±0.004b	0.035±0.004c	0.038±0.001c	0.037±0.006c	0.045±0.004b	0.089±0.006a
47	Alpha-muurolol	19435-97-3	herbal, honey	0.004±0e	0.008±0d	0.052±0.001b	0.008±0d	0.013±0c	0.059±0.003a
48	2-Hexadecanol	14852-31-4	null	0.02±0.001d	0.027±0.004c	0.033±0.001b	0.032±0.005bc	0.033±0.001b	0.055±0.002a
49	Farnesol	4602-84-0	mild, fresh, sweet	0.001±0d	0.004±0c	0.004±0c	0.003±0c	0.027±0.002a	0.018±0.001b
50	Octadecanol	112-92-5	bland	0.007±0d	0.009±0.001c	0.008±0c	0.007±0.001d	0.01±0b	0.076±0.006a
				**Aldehydes**					
51	Benzaldehyde	100-52-7	bitter, almond, burnt, sugar	2.476±0.008a	0.779±0.049d	0.706±0.015e	0.941±0.019c	1.117±0.083b	0.827±0.004d
52	Octanal	124-13-0	citrus, fat, green, oil	0.248±0.021e	0.493±0.02d	1.668±0.048b	1.67±0.06b	1.815±0.114a	0.598±0.009c
53	Benzeneacetaldehyde	122-78-1	berry, geranium, honey	22.94±1.622a	17.264±0.954b	17.634±1.613b	17.076±1.544b	17.876±0.637b	14.238±0.848c
54	(2E,6Z)-nona-2,6-dienal	557-48-2	cucumber, green, wax	2.6±0.039a	1.188±0.082b	1.102±0.015bc	1.01±0.077c	1.12±0.089bc	1.03±0.097c
55	Decanal	112-31-2	floral, fried, orange, peel	4.56±0.029a	3.051±0.181d	3.649±0.086b	3.772±0.206b	3.351±0.053c	2.659±0.095e
56	β-Cyclocitral	432-25-7	herbal, rose, sweet	8.672±0.566a	8.313±0.812a	8.08±0.251a	8.113±0.111a	8.007±0.416a	6.384±0.344b
57	(Z)-citral	106-26-3	sweet, lemon, peel	4.546±0.268a	3.905±0.097b	4.813±0.099a	4.526±0.173a	4.763±0.205a	4.604±0.572a
58	(2E)-2-Decenal	3913-81-3	fat, fish, orange	1.195±0.031a	1.272±0.032a	0.993±0.014b	0.866±0.073c	0.703±0.048d	0.945±0.077bc
59	(E)-citral	5392-40-5	sharp, lemon, sweet	10.318±0.372a	8.099±0.274b	7.746±0.212bc	7.042±0.048d	7.505±0.52cd	5.325±0.256e
60	(E)-2-Hexenal	6728-26-3	green, banana, aldehydic	21.485±0.232b	23.643±1.893ab	25.067±2.177a	24.351±1.113a	25.081±1.567a	22.594±1.593ab
61	(E)-2-Octenal	2548-87-0	dandelion, fat, fruit	1.871±0.081b	2.245±0.013a	1.861±0.044b	1.814±0.165b	1.896±0.019b	1.914±0.052b
62	(2E)-2-Nonenal	18829-56-6	fatty, green, cucumber	2.543±0.02a	1.594±0.019b	1.211±0.041d	1.258±0.059cd	1.304±0.024c	0.809±0.026e
63	10-Undecenal	112-45-8	waxy, aldehydic, rose	0±0b	0±0b	0±0b	0±0b	0.401±0.006a	0±0b
64	Pentanal	110-62-3	almond, bitter, malt	0.071±0.003c	0.057±0.006d	0.083±0.007b	0.119±0.017a	0.056±0.007d	0.056±0.006d

65	(E)-2-Methylbut-2-enal	497-03-0	strong, green	0.431±0.024a	0.463±0.018a	0.359±0.019b	0.364±0.027b	0.158±0.003c	0.117±0.002d
66	2-Methyl-2-butenal	1115-11-3	pungent, green	0.1±0.011a	0.074±0.005b	0.048±0.002d	0.038±0.002e	0.06±0.002c	0.024±0.001f
67	(E)-2-Pentenal	1576-87-0	pungent, green	0.506±0.015a	0.521±0.017a	0.483±0.046a	0.378±0.021b	0.329±0.005c	0.14±0.025d
68	3-methylbut-2-enal	107-86-8	sweet, fruity	0.381±0.007a	0.32±0.008b	0.334±0.007b	0.314±0.061b	0.143±0.018c	0.049±0.006d
69	3-Methyl-2-butenal	107-86-8	almond, roasted	2.546±0.031a	2.011±0.132b	1.351±0.039c	1.399±0.098c	0.475±0.022d	0.204±0.017e
70	Hexanal	66-25-1	green, banana, fatty, cheesy	0.707±0.051c	0.691±0.028c	0.746±0.027c	0.906±0.121b	0.961±0.037b	1.176±0.095a
71	Cyclopentanecarbaldehyde	872-53-7	null	0.015±0e	0.027±0.001d	0.031±0c	0.068±0.002a	0.062±0.002b	0.067±0.004a
72	2-hexenal	505-57-7	sweet, almond, fruity	0.96±0.01b	1.086±0.055a	1.097±0.056a	1.073±0.012a	0.114±0.015c	0.099±0.006b
73	Heptanal	111-71-7	citrus, fat, green	0.186±0.004a	0.139±0.014b	0.16±0.012b	0.138±0.014b	0.06±0.006c	0.06±0.004c
74	Methional	3268-49-3	cooked, potato, soy	0±0e	0.052±0.003c	0.088±0.002a	0.063±0b	0.03±0.001d	0.036±0.003d
75	(E,E)-2,4-Hexadienal	142-83-6	sweet, green, spicy	0.24±0.028bc	0.315±0.054a	0.267±0.007ab	0.227±0.03bc	0.239±0.011bc	0.205±0.021bc
76	(E,E)-2,4-Heptadienal	4313-03-5	fat, nut	0.33±0.006b	0.16±0.014c	0.121±0.014d	0.298±0.012b	0.232±0.016b	0.384±0.048a
77	Citronellal	106-23-0	citrus, fat, leaf	0.113±0.012c	0.121±0.006c	0.174±0.006b	0.29±0.019a	0.157±0.012b	0.151±0.008b
78	Dodecanal	112-54-9	citrus, fat, lily	0.064±0.002d	0.073±0.003c	0.102±0.005a	0.076±0.001c	0.086±0.006b	0.089±0.01b
79	(Z)-9-tetradecenal	53939-27-8	null	0.017±0.001c	0.02±0.002b	0.03±0.003a	0.024±0.002b	0.028±0.001a	0.031±0.004a
80	(E)-2-Hexadecenal	22644-96-8	null	0.001±0c	0.003±0b	0.002±0b	0.003±0b	0.001±0c	0.046±0.002a
				**Ketones**					
81	Jasmone	488-10-8	woody, herbal, floral	6.824±0.237c	8.296±0.183ab	8.662±0.782a	8.55±0.303ab	7.929±0.287b	6.192±0.341c
82	β-ionone	79-77-6	floral, violet	7.437±0.332a	6.339±0.192b	5.948±0.047c	5.575±0.247cd	5.565±0.138d	5.865±0.187cd
83	β-Ionone epoxide	23267-57-4	fruit, wood	1.7±0.143a	1.399±0.081bc	1.406±0.006b	1.279±0.016cd	1.246±0.026d	1.702±0.034a

84	β-damascenone	23726-93-4	apple, rose, honey	7.997±0.346a	5.285±0.317c	5.914±0.015b	5.606±0.2bc	5.442±0.191c	5.324±0.022c
85	α-ionone	127-41-3	violet, wood	0.964±0.073ab	0.883±0.05b	1.039±0.065a	0.903±0.056b	0.906±0.01b	0.748±0.063c
86	Neryl acetone	3879-26-3	fatty, metallic	0.782±0.016d	0.864±0.021d	1.619±0.098a	0.983±0.021c	1.011±0.106c	1.166±0.048b
87	3-Penten-2-one	625-33-2	fruity, fishy	0.073±0.001a	0.034±0.003c	0.028±0.001d	0.045±0.006b	0.025±0.002e	0.022±0.001e
88	2-Cyclopenten-1-one	930-30-3	null	1.229±0.068c	1.565±0.068a	1.58±0.025a	1.641±0.185a	1.329±0.073b	1.143±0.119c
89	6-Methylhept-5-en-2-one	110-93-0	citrus, mushroom, pepper	0.268±0.027b	0.324±0.02a	0.18±0.023c	0.149±0.007d	0.119±0.003e	0.064±0.004f
90	Vinylpyrrolidinone	88-12-0	null	0.879±0.004d	0.212±0.008e	2.238±0.175b	1.99±0.084c	3.016±0.011a	2.239±0.087b
91	4-Isopropylcyclohexanone	5432-85-9	null	0.017±0.001b	0.019±0.001b	0.018±0.001b	0.01±0.002c	0.018±0.001b	0.042±0.002a
92	3-ethyl-4-methylpyrrole-2,5-dione	20189-42-8	null	0.019±0.002d	0.085±0.005c	0.114±0.006b	0.124±0.011b	0.185±0.01a	0.163±0.018a
93	Geranylacetone	3796-70-1	fresh, fruity, waxy	0.105±0.012c	0.143±0.008b	0.142±0.011b	0.223±0.018a	0.09±0.009c	0.026±0.002d
94	beta-Dihydro-ionone	17283-81-7	earthy, woody, orris	0.129±0.014a	0.076±0c	0.106±0.003b	0.02±0.002f	0.027±0.001e	0.036±0.001d
95	2,5-Di-tert-butyl-1,4-benzoquinone	2460-77-7	null	0.048±0.004a	0.024±0.003b	0.019±0.002c	0.028±0.001b	0.03±0.004b	0.027±0b
		**Acids and esters**							
96	Hexanoic acid	142-62-1	cheese, oil, pungent, sour	4.133±0.269a	3.013±0.296cd	3.209±0.092bc	3.425±0.081b	3.093±0.031bc	2.793±0.274d
97	Geranic acid	459-80-3	green, moldy, woody	12.153±0.568a	9.963±0.157b	10.013±0.237b	10.282±0.581b	10.415±0.236b	9.939±0.947b
98	Methyl 2-methoxybenzoate	606-45-1	floral	1.065±0.008a	0.983±0.052b	0.925±0.012c	0.79±0.016d	0.756±0.017d	0.69±0.014e
99	2-(2-Butoxyethoxy)ethyl acetate	124-17-4	null	2.191±0.141a	2.27±0.163a	2.421±0.248a	2.412±0.125a	2.144±0.135a	2.395±0.094a
100	δ-Decalactone	705-86-2	coconut	0.788±0.026a	0.385±0.032c	0.453±0.01b	0.37±0.011c	0.279±0.002d	0.235±0.003e
101	Dihydroactinidiolide	17092-92-1	musk, coumarin	0.85±0.058a	0.708±0.042b	0.775±0.019ab	0.759±0.06b	0.717±0.044b	0.774±0.015ab
102	Geranyl isovalerate	109-20-6	apple, fruit, rose	0±0b	0±0b	0±0b	0±0b	0.421±0.041a	0±0b

103	Methyl salicylate	119-36-8	almond, caramel, peppermint	14.936±1.107d	15.568±0.064cd	17.382±1.288bc	20.408±1.553a	18.008±1.353b	15.738±0.297cd
104	(E)-3-hexenoic acid	1577-18-0	powerful, fruity, honey	3.536±0.074c	7.435±0.197b	11.92±0.499a	11.463±0.642a	11.557±0.192a	11.609±0.215a
105	2-Ethyl-3-hydroxyhexyl 2-methylpropanoate	74367-31-0	null	1.63±0.167c	1.593±0.03c	1.533±0.06c	1.631±0.028c	1.773±0.028b	2.339±0.065a
106	(Z)-3-Hexenyl butyrate	16491-36-4	fresh, apple, wine	1.066±0.086e	1.192±0.093d	2.166±0.035c	2.43±0.076b	3.363±0.063a	1.199±0.027d
107	linalyl formate	115-99-1	citrus, coriander	4.209±0.07cd	6.099±0.24a	4.395±0.099c	4.974±0.323b	4.252±0.407cd	3.902±0.244d
108	Isopentyl 3-hydroxy-2-methylenebutanoate	80758-72-1	null	2.537±0a	0.221±0.021c	0.239±0.03c	0.589±0.051b	0.579±0.009b	0±0d
109	Menthyl acetate	89-48-5	mint, cool	2.495±0.074a	2.074±0b	2.363±0.29a	1.195±0.115c	1.438±0.142c	1.071±0.168d
110	Geranyl formate	105-86-2	floral	1.148±0.087a	0.937±0.021b	0.622±0.012cd	0.602±0.002d	0.627±0.042cd	0.678±0.03c
111	(3Z)-3-Hexen-1-yl hexanoate	31501-11-8	fruit, prune	2.444±0.197d	2.906±0.051c	3.944±0.054a	3.607±0.109b	3.897±0.145a	2.516±0.106d
112	Methyl 4-methyl valerate	2412-80-8	fruit	0.011±0.001b	0.014±0.001a	0.008±0.001c	0.01±0.001b	0.009±0b	0.016±0.002a
113	Isopentyl acrylate	4245-35-6	null	0.074±0.007d	0.059±0.006e	0.08±0.004d	0.108±0.013c	1.711±0.105a	0.251±0.027b
114	2-hexen-4-olide	2407-43-4	spice	0.012±0d	0.013±0d	0.027±0.001c	0.047±0.008b	0.068±0.011a	0.062±0.003a
115	(E)-3-hexen-1-yl acetate	3681-82-1	fruit	0.054±0.004d	0.079±0.011c	0.262±0.021a	0.158±0.027b	0.043±0.001e	0.021±0.003f
116	γ-Caprolactone	695-06-7	herbal, coconut, sweet	0.798±0.078a	0.078±0.01f	0.122±0.011e	0.237±0.013c	0.37±0.039b	0.156±0.005d
117	Allyl hexanoate	123-68-2	pineapple	0.399±0.048b	0.304±0.01c	0.41±0.003b	0.414±0.04b	0.457±0.052a	0.242±0.018d
118	N-heptanoic acid	111-14-8	apricot, floral, sour	0.197±0.008a	0.135±0.006b	0.104±0.01c	0.095±0.008cd	0.095±0.003d	0.009±0.001e
119	δ-Octalactone	698-76-0	coconut, peach	0.04±0.005d	0.089±0.01b	0.055±0.008c	0.056±0.008c	0.09±0.009b	0.145±0.005a
120	Decanoic acid	334-48-5	dust, fat, grass	0.209±0.013b	0.208±0.01b	0.161±0.004c	0.156±0.008c	0.154±0.015c	0.353±0.026a
121	Ethyl 9-decenoate	67233-91-4	fruity, fatty	0.045±0.002a	0.037±0.004b	0.02±0.002c	0.019±0.003d	0.04±0.005ab	0.043±0.006ab
122	Methyl 9-oxononanoate	1931-63-1	null	0.012±0.001d	0.026±0c	0.055±0.01a	0.036±0b	0.035±0.001b	0.025±0.002c

123	Dimethyl phthalate	131-11-3	odorless	0.914±0.016c	0.718±0.035d	1.226±0.08a	1.096±0.091b	0.77±0.041d	0.737±0.059d
124	(Z)-8-Dodecenyl acetate	28079-04-1	null	0.002±0e	0.008±0d	0.014±0.001b	0.01±0.001c	0.016±0.001b	0.05±0.006a
125	Methyl 4-heptylbenzoate	6892-80-4	null	0.011±0.001d	0.012±0.001d	0.017±0.001c	0.013±0.002d	0.041±0.002b	0.053±0.002a
126	Trans-2-tetradecenal	51534-36-2	citrus, waxy, fatty	0.015±0.002d	0.023±0c	0.04±0.004b	0.044±0.004b	0.045±0.004b	0.069±0.004a
127	Myristic acid	544-63-8	burnt, cheese, harsh, oil	0.004±0d	0.005±0.001c	0.005±0c	0.017±0b	0.003±0e	0.034±0.003a
128	Isopropyl myristate	110-27-0	faint, oily, fatty	0.01±0.001d	0.033±0.002c	0.035±0.003c	0.09±0.016a	0.08±0.004b	0.084±0.007a
129	Diisobutyl phthalate	84-69-5	null	0.05±0.003c	0.085±0.003b	0.078±0.007b	0.082±0.004b	0.134±0.017a	0.109±0.009a
130	Dibutyl phthalate	84-74-2	faint, odor	0.129±0.01e	0.224±0.011a	0.196±0.006b	0.17±0.008c	0.177±0.002c	0.156±0.006d
131	Palmitic acid	57-10-3	slightly, waxy, fatty	0.052±0.006c	0.074±0.003b	0.061±0.007c	0.06±0.007c	0.079±0.007b	0.252±0.018a
				**Hydrocarbons**					
132	β-Myrcene	123-35-3	balsamic, fruit, herb	14.135±0.942c	14.271±0.11bc	15.549±1.218ab	14.955±0.475abc	15.977±0.789a	15.464±0.595abc
133	Tridecane	629-50-5	null	2.997±0.148a	2.959±0.192a	2.776±0.133a	2.484±0.192b	2.494±0.02b	1.671±0.171c
134	Tetradecane	629-59-4	mild, waxy	1.352±0.062c	1.742±0.329abc	1.867±0.058a	1.642±0.18abc	1.767±0.134ab	1.456±0.367bc
135	Hexadecane	544-76-3	null	0.501±0.007bc	0.519±0.012b	0.559±0.025a	0.477±0.011c	0.423±0.012d	0.433±0.024d
136	Dodecane	112-40-3	alkane	1.507±0.074c	1.624±0.024bc	1.93±0.046a	1.776±0.147ab	1.477±0.107c	0.755±0.071d
137	β-Caryophyllene	87-44-5	fried, spice, wood	0±0c	0.762±0a	0.714±0.007b	0±0c	0±0c	0±0c
138	Cis-β-Farnesene	28973-97-9	boiled, vegetable, floral	1.182±0.154a	0.77±0.046c	0.93±0.019b	0.795±0.04c	0.762±0.057c	0.778±0.038c
139	α-Farnesene	502-61-4	boiled, vegetable, floral	0.758±0.074a	0.631±0.013b	0.564±0.007c	0.365±0.007d	0.412±0.016d	0.373±0.004d
140	(Z,E)-alpha-farnesene	26560-14-5	boiled, vegetable, floral	0±0c	0.286±0.022b	0.38±0.032a	0±0c	0±0c	0±0c
141	Epi-Cubebol	38230-60-3	null	0±0c	0±0c	0±0c	0.404±0.005b	0.494±0.024a	0±0c

142	β-himachalene	1461-03-6	null	0.298±0.003a	0.172±0.015b	0.183±0.01b	0.141±0.004c	0.143±0.007c	0.102±0.006d
143	2,3,5-Trimethylhexane	1069-53-0	null	1.624±0.042c	2.005±0.004a	1.808±0.095b	1.369±0.062d	0.74±0.09e	0.296±0.047f
144	2,4-Dimethylheptane	2213-23-2	null	0.368±0.029a	0.293±0.006c	0.328±0.035ab	0.32±0.001b	0.13±0.017d	0.082±0.002e
145	styrene	100-42-5	sweet, balsam, floral	0.095±0.007a	0.11±0.011a	0.098±0.011a	0.108±0.01a	0.068±0.004b	0.04±0.003c
146	Dihydromyrcene	2436-90-0	herbal, citrus	0.047±0.004f	0.061±0.004e	0.169±0.006d	0.3±0.033a	0.25±0.003b	0.216±0.008c
147	Trans-4-decene	19398-89-1	null	0.223±0.011b	0.269±0.028a	0.255±0.023a	0.097±0.005c	0.066±0.005d	0.045±0.006e
148	(6E)-2,6-dimethylocta-2,6-diene	2792-39-4	null	0.643±0.068b	0.596±0.053b	0.673±0.063b	0.945±0.11a	0.267±0.016c	0.202±0.025d
149	β-pinene	127-91-3	woody, resinous, pine	0.175±0.011d	0.169±0.018d	0.235±0.012c	0.249±0.006c	0.349±0.029b	0.464±0.017a
150	3-(2-Methylpropyl)-1-cyclohexene	4104-56-7	null	0.17±0.007e	0.695±0.002a	0.54±0.008b	0.339±0.004c	0.301±0.003d	0.336±0.043cd
151	Limonene	138-86-3	citrus, herbal, camphor	0.158±0.022e	0.199±0.027d	0.28±0.03b	0.249±0.025c	0.425±0.047a	0.316±0.033b
152	Pentylcyclopentane	3741-00-2	null	0.474±0.024c	0.141±0.007e	0.677±0.003a	0.648±0.02b	0.529±0.066c	0.305±0.007d
153	2-methyldecane	6975-98-0	null	0.757±0.077a	0.614±0.016b	0.329±0.02d	0.33±0.009d	0.362±0.021c	0.139±0.003e
154	Terpinolene	586-62-9	fresh, woody, sweet	0.132±0.003c	0.086±0.005e	0.208±0.01a	0.174±0.001b	0.106±0.005d	0.093±0.004e
155	1-Undecyne	2243-98-3	null	0.269±0.004a	0.251±0.003b	0.25±0.014b	0.244±0.022b	0.132±0.018d	0.192±0.023c
156	(3E,5E)-2,6-Dimethyl-1,3,5,7-octatetracene	460-01-5	null	0.024±0.002d	0.074±0.006c	0.068±0.002c	0.094±0.007b	0.098±0.009b	0.147±0.016a
157	2,6,11-Trimethyldodecane	31295-56-4	null	0.509±0.007a	0.46±0.035b	0.354±0.013d	0.408±0.016c	0.326±0.02d	0.427±0.054bc
158	2-decyloxirane	2855-19-8	null	0.068±0.002b	0.058±0.006c	0.071±0.008ab	0.069±0.006ab	0.064±0.004bc	0.075±0.003a
159	(+)-Longifolene	475-20-7	sweet, woody, rose, medical	0.013±0.001c	0.009±0e	0.024±0.002a	0.012±0d	0.017±0.002b	0.028±0.002a
160	2-methyltetradecane	1560-95-8	null	0.253±0.016b	0.202±0.004c	0.29±0.023ab	0.268±0.008b	0.252±0.011b	0.297±0.009a
161	Pentadecane	629-62-9	waxy	0.06±0.004b	0.047±0.006c	0.051±0.004c	0.057±0.005b	0.051±0.003c	0.075±0.004a

162	2,6,10-trimethyltetradecane	14905-56-7	null	0.052±0.002	0.051±0.003	0.063±0.001	0.051±0.003	0.051±0.003	0.114±0.013
163	2,6,10,14-Tetramethylpentadecane	1921-70-6	null	0.024±0.001	0.038±0.002	0.041±0.005	0.037±0.004	0.033±0.002	0.079±0.007
164	1,2-Epoxyhexadecane	7320-37-8	null	0.017±0.002c	0.025±0b	0.034±0.002a	0.032±0.004a	0.03±0.002a	0.036±0.004a
165	Heptadecane	629-78-7	null	0.019±0.001f	0.029±0.001e	0.039±0.002c	0.033±0.002d	0.061±0.002b	0.075±0.01a
166	Octadecane	593-45-3	null	0.013±0.001e	0.022±0.001d	0.028±0.004c	0.037±0.006c	0.078±0a	0.057±0.004b
167	Nonadecane	629-92-5	bland	0.021±0.002d	0.031±0.002c	0.17±0.005a	0.018±0.002d	0.06±0.006b	0.03±0c
168	1,2-Epoxyoctadecane	7390-81-0	null	0.007±0e	0.01±0.001c	0.012±0.002c	0.008±0.001d	0.031±0.003a	0.019±0.001b
169	Icosane	112-95-8	waxy	0.004±0c	0.002±0d	0.017±0.001a	0.012±0.001b	0.019±0.001a	0.013±0.001b
				**Others**					
170	Indole	120-72-9	burnt, mothball	0±0d	1.166±0.032b	1.49±0.194a	0.864±0.01c	0.795±0.083c	0±0d
171	2,6,10,10-Tetramethyl-1-oxaspiro[4.5]dec-6-ene	36431-72-8	honey	3.417±0.024a	3.153±0.04b	3.093±0.137b	2.834±0.179c	2.252±0.212d	1.547±0.132e
172	2-Methyltetrahydrofuran	96-47-9	null	1.024±0.087b	1.113±0.101ab	1.123±0.055a	1.073±0.103b	0.734±0.007c	0.454±0.037d
173	3,4-Dihydro-2H-pyran	110-87-2	null	0.126±0.003c	0.141±0.008b	0.156±0.003a	0.155±0.012ab	0.112±0.003d	0.104±0.013d
174	3-methyl-4,5-dihydrofuran	34314-83-5	null	0.361±0.032a	0.141±0.007e	0.279±0.029b	0.212±0.008c	0.195±0.005d	0.089±0.003f
175	2-Ethylpyrazine	13925-00-3	burnt, green	0.05±0.004c	0.103±0.006b	0.107±0.001b	0.114±0.009b	0.165±0.007a	0.156±0.008a
176	1-ethylpyrrole-2-carbaldehyde	2167-14-8	burnt, roasted, smoky	0.856±0.115c	1.843±0.063a	2.027±0.013a	2.048±0.216a	1.607±0.056b	1.553±0.232b
177	Caryophyllene Oxide	1139-30-6	herb, must, spice	0.004±0e	0.026±0.003c	0.032±0.002b	0.03±0.003bc	0.091±0.013a	0.006±0d
178	Diosphenol	490-03-9	minty, black, currant	0.157±0.012a	0.121±0.005b	0.103±0.006c	0.103±0.004c	0.108±0.004c	0.066±0.001d
179	Butylated hydroxytoluene	128-37-0	toasted, cereal	0.045±0.003d	0.085±0.004c	0.119±0.014a	0.117±0.016a	0.111±0.006a	0.097±0.008b
180	2,5-Di-tert-butylphenol	5875-45-6	null	0.085±0.001b	0.031±0.001e	0.035±0.002d	0.034±0.003de	0.044±0.002c	0.178±0.014a

Figures S1. Aromatic amino acid content of black tea with different degrees of sun withering.

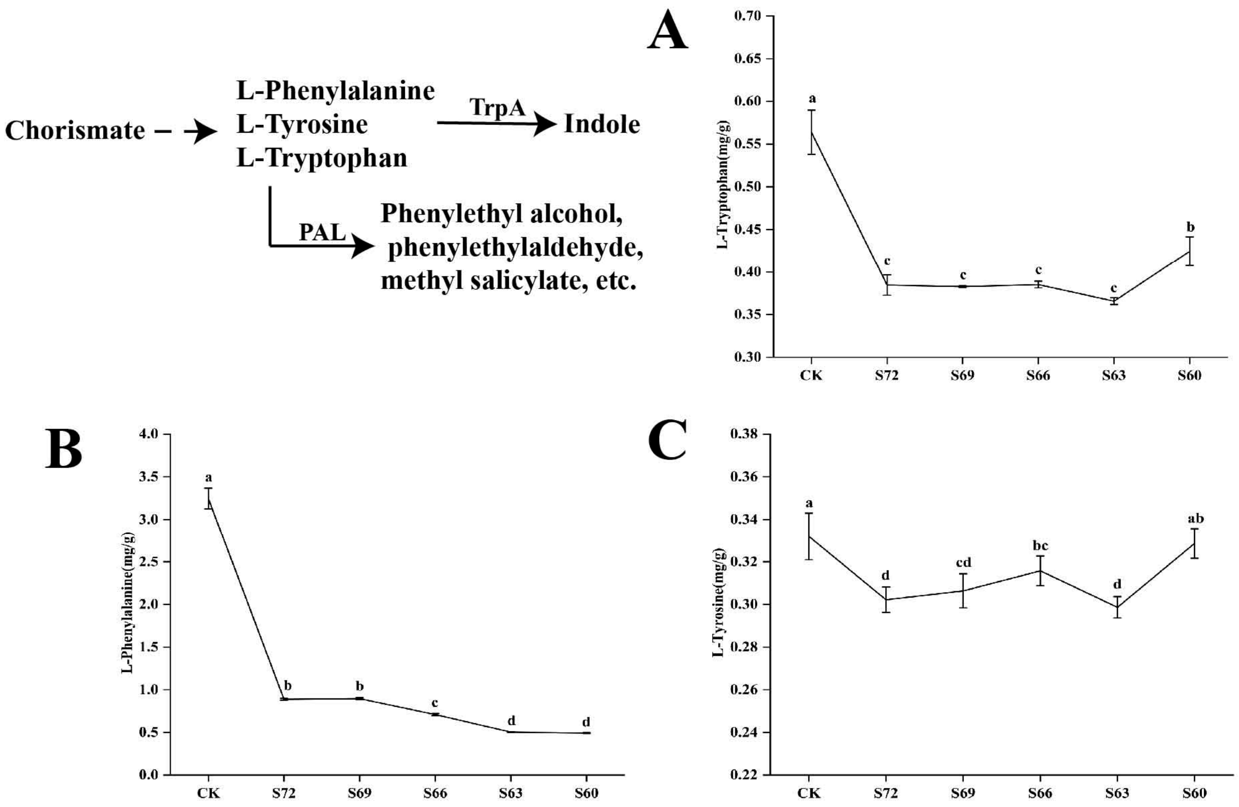

Note: (A) L-tryptophan. (B) L-phenylalanine. (C) L-tyrosine. Trp A: α-branched-chain tryptophan synthase, PAL: phenylalanine deaminase. Different lowercase letters on the curve indicate significant differences between groups at $P<0.05$ level.

Figure S2. Key volatiles content of black tea with different degrees of sun withering.

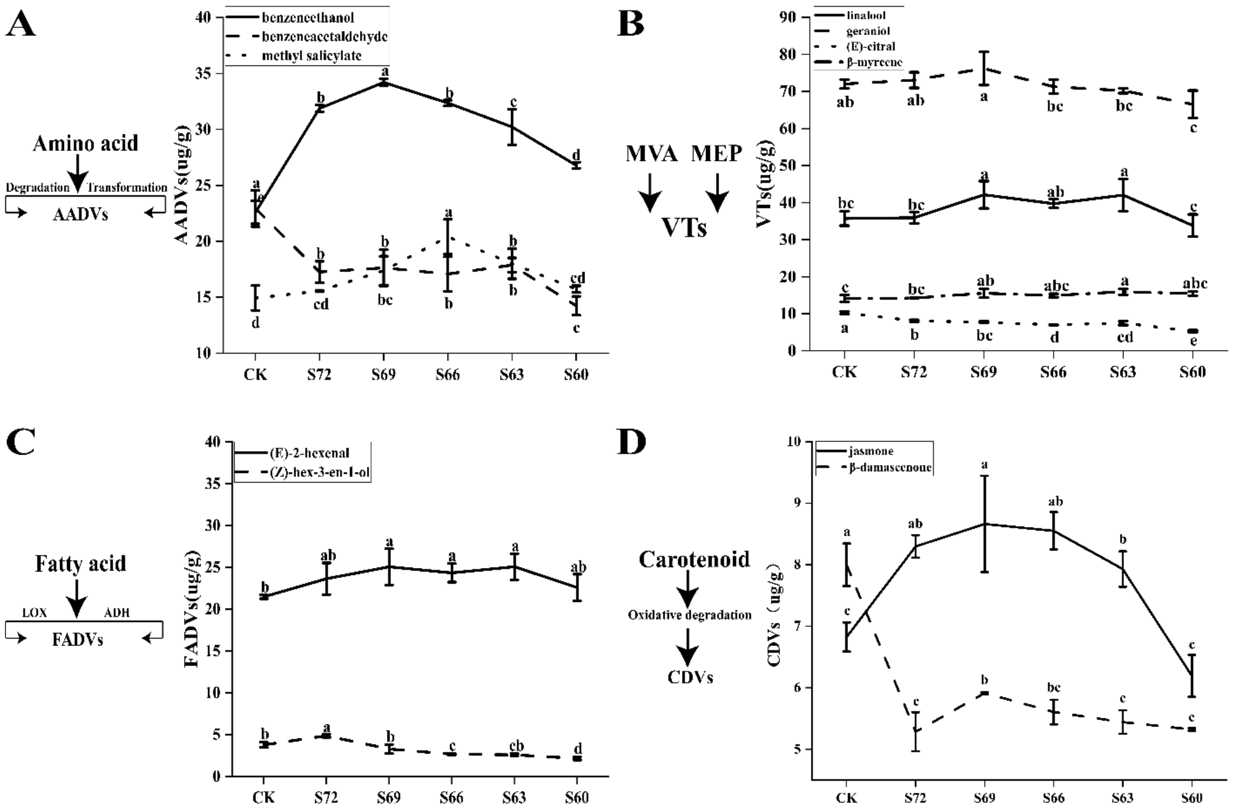

Note: (A) AADVs volatiles. (B) VTs volatiles. (C) FADVs volatiles. (D) CDVs volatiles. MVA: Mevalonate pathway. MEP: methylerythritol phosphate pathway. LOX: Lipoxygenase. ADH: Alcohol dehydrogenase. Different lowercase letters on the curves indicate significant differences between groups at $P<0.05$ level.
